# All‐Trans Retinoic Acid Inhibits Canine Influenza Virus Replication Through Keratin 27–Mediated Autophagy Modulation

**DOI:** 10.1155/tbed/2088414

**Published:** 2026-10-22

**Authors:** Qi Li, Shaotang Ye, Qi Jiang, Benqiang Li, Jinghua Cheng, Jie Tao, Ying Shi, Pan Tang, Huili Liu

**Affiliations:** ^1^ Institute of Animal Science and Veterinary Medicine Shanghai Academy of Agricultural Sciences Shanghai 201106 China saas.sh.cn; ^2^ Shanghai Key Laboratory of Agricultural Genetic Breeding Shanghai 201106 China; ^3^ College of Veterinary Medicine South China Agricultural University Guangzhou 510642 China scau.edu.cn; ^4^ Department of Veterinary Medicine College of Animal Sciences Zhejiang University Hangzhou 310058 China zju.edu.cn

**Keywords:** all-trans retinoic acid, autophagy, influenza A virus, keratin 27

## Abstract

The influenza A virus (IAV) poses a significant threat to public health. Thus, antiviral drug research is a hotspot for IAV. All‐trans retinoic acid (ATRA), a bioactive metabolite of vitamin A, exhibits different effects on different subtypes of IAV. In this study, ATRA was found to inhibit canine influenza virus (CIV) replication in Madin–Darby canine kidney (MDCK) and mink lung cells. Furthermore, the transcriptomic analysis revealed that ATRA significantly upregulated keratin 27 (KRT27), a cytoskeletal protein not previously associated with antiviral immunity. Overexpression of KRT27 suppressed CIV replication, while its knockdown enhanced viral load. Mechanistically, ATRA and KRT27 promoted autophagy, counteracting CIV‐mediated autophagy hijacking. Notably, ATRA’s antiviral effect was species‐specific, showing no inhibition in human BEAS‐2B or A549 cells. These findings identify ATRA‐induced KRT27 as a novel regulator of autophagy and innate immunity against CIV, offering a potential host‐directed therapeutic strategy against influenza viruses.

## 1. Introduction

Influenza A virus (IAV) remains a global respiratory pathogen that causes annual epidemics and sporadic pandemics with substantial morbidity and mortality. The segmented negative‐sense RNA genome of IAV encodes up to 18 proteins, including surface glycoproteins hemagglutinin (HA), neuraminidase (NA), matrix protein 1 (M1), and matrix protein 2 (M2), that drive receptor binding, viral assembly, and virion release, respectively, while internal proteins such as nucleoprotein (NP) and nonstructural protein 1 (NS1) subvert host antiviral responses [1–4]. Rapid antigenic drift and occasional reassortment events challenge vaccine efficacy, compelling the development of host‐directed therapeutics. Accumulating evidence suggests that IAV replication is closely linked to cellular homeostatic pathways, among which lipid metabolism, redox balance, and autophagy are emerging as key determinants of viral fitness [5–7]. Understanding how these cellular pathways can be pharmacologically manipulated is therefore a prerequisite for next‐generation antiviral strategies.

All‐trans retinoic acid (ATRA), the principal bioactive metabolite of vitamin A, transduces genomic and nongenomic signals through nuclear retinoic acid receptors (RARα/β/γ) and retinoid X receptors (RXRs) [8, 9]. Beyond its canonical roles in embryogenesis and epithelial differentiation, ATRA has recently been recognized as a potent modulator of innate and adaptive immunity, including the induction of type I interferons, polarization of alveolar macrophages, and enhancement of mucosal IgA responses [10–12]. Importantly, ATRA can be recycled endogenously in lung tissue, achieving micromolar concentrations sufficient to regulate metabolic and inflammatory gene sets. Several independent studies have demonstrated that ATRA restricts the replication of RNA viruses such as SARS‐CoV‐2 and HBV by perturbing membrane fluidity required for viral entry and by destabilizing viral proteins [13, 14]. However, the effect of ATRA on IAV life cycle remains controversial, with reports ranging from suppression of viral transcription to exacerbation of inflammation‐mediated lung pathology.

Autophagy is an evolutionarily conserved catabolic program that sequesters cytoplasmic cargo—damaged organelles, protein aggregates, or invading pathogens—within double‐membrane autophagosomes for lysosomal degradation [15, 16]. The process is executed by more than 40 autophagy‐related (ATG) proteins and is negatively gated by the PI3K–AKT–mTORC1 axis while positively regulated by AMPK and Beclin‐1/Vps34 complexes. Selective autophagy receptors such as p62/SQSTM1, NDP52, and OPTN deliver ubiquitinated cargo to LC3‐positive autophagosomes, thereby bridging innate immunity to intracellular quality control [16–19]. Mounting evidence reveals that IAV actively hijacks autophagic membranes for viral trafficking and that pharmacological enhancement of autophagy flux curtails viral progeny release [20–22]. Determining whether ATRA regulates this delicate balance toward an antiviral or cytopathic outcome is therefore essential for repurposing retinoids and retinoic acids against influenza.

In this study, we confirmed that ATRA inhibits canine influenza virus (CIV) replication in a dose‐dependent and cell species–dependent manner. Transcriptomic analysis revealed that ATRA induces significant expression of the keratin 27 (KRT27) gene, which in turn inhibits CIV replication and is associated with cellular autophagy. Therefore, our findings provide a basis for ATRA’s inhibition of mammalian (canine) H3N2 influenza viruses and offer important data for the development of novel therapeutic strategies against influenza viruses.

## 2. Materials and Methods

### 2.1. Cell, Virus, and Reagents

Madin–Darby canine kidney (MDCK) cells, human normal lung epithelial cells (BEAS‐2B), and human nonsmall cell lung cancer cells (A549) were cultured at 37°C and 5% (v/v) CO_2_ in Dulbecco’s modified Eagle medium (DMEM; Gibco, USA) and 10% fetal bovine serum (FBS; Gibco). Mink lung epithelial cells (Mv.1.Lu) were cultured in modified Eagle medium (MEM; Gibco). CIV H3N2 (A/canine/Guangdong/02/2011, C/GD/02) was propagated in specific pathogen–free chick embryos, and the virus was titrated as log_10_ (median tissue culture infectious dose [TCID_50_]/mL) and stored at −80°C. The reagents ATRA, rapamycin, chloroquine, and CCK‐8 kit were purchased from Topscience (China).

### 2.2. Evaluation of Cytotoxicity and Antiviral Efficacy of ATRA

Cells were seeded in 96/12/6‐well plates and grown in DMEM with 2% FBS. The confluent monolayer cells were pretreated with ATRA for the indicated time‐course. (a) For cytotoxicity experiments, cell viability was determined by the CCK‐8 kit. (b) For antiviral efficacy experiments, cells were infected with CIV at the indicated multiplicity of infection (MOI). After 1 h of virus inoculation, the cells were washed with phosphate‐buffered saline (PBS, Gibco) three times and replenished with fresh DMEM containing 2% FBS. The antiviral efficacy was determined by indirect immunofluorescence analysis (IFA), virus titration, quantitative real‐time PCR (qPCR), and western blotting.

### 2.3. Plasmids and Antibodies

The full‐length canine KRT27 gene was inserted into the mammalian expression plasmid pCAGGS‐HA using the ClonExpress Ultra One Step Cloning Kit V3 (Vazyme, China). Primary and secondary antibodies used in this study are listed in Supporting Information 1: File 10.

### 2.4. Indirect IFA

MDCK cells were treated with or without ATRA for the indicated time course. Before fixation, the cells were washed 3 times with PBS. The cells were fixed in cold 4% paraformaldehyde at room temperature for 10 min. For immunofluorescence labeling, cells were blocked for 10 min at room temperature (QuickBlock Blocking Buffer for Immunol Staining, Beyotime, Shanghai, China) and then incubated overnight at 4°C with the primary antibody and for 1 h at room temperature with the secondary antibody; nuclei were stained with 4′, 6‐diamidino‐2‐phenylindole (Beyotime) for 5 min. The fluorescence signal was observed with a confocal laser scanning microscope (SP8 TCS; Leica, Germany).

### 2.5. qPCR

The cDNA was synthesized by HiScript IV All‐in‐One Ultra RT SuperMix for qPCR (Vazyme). The qPCR was carried out on a LightCycler 480 (Roche, Switzerland) with ChamQ SYBR Color qPCR Master Mix (Vazyme) using the following program: 95°C for 30 s, followed by 40 cycles of 95°C for 10 s and 60°C for 30 s. Samples were analyzed in triplicate, and three independent experiments were performed. The indicated mRNA expression to glyceraldehyde 3‐phosphate dehydrogenase (GAPDH) was calculated with the 2^−∆∆Ct^ method and plotted as fold change compared to mock‐transfected cells. Primers used for qPCR were designed based on published sequences and are listed in Supporting Information 1: File 10.

### 2.6. Western Blotting

MDCK cells were seeded and transfected with or without plasmids. Then, the cells were infected with CIV (or left uninfected) 24 h after 24 h transfection. The cells were lysed using RIPA Lysis and Extraction Buffer (Thermo Scientific, USA). Lysates were collected and centrifuged for 15 min at 15,000 rpm. A total of 30 μg of each sample was separated by sodium dodecyl sulfate polyacrylamide gel electrophoresis and transferred to a nitrocellulose membrane that was blocked for 15 min with QuickBlock Blocking Buffer for Western Blot (Beyotime) and then incubated overnight at 4°C with primary antibodies, followed by a 1 h incubation with secondary antibody. Protein bands were visualized by Odyssey Sa (Li‐cor, USA).

### 2.7. RNA Interference

Small interfering RNA targeting the different fragments of the canine KRT27 gene was prepared. The siRNAs and a scrambled negative control siRNA were synthesized by RiboBio (siBDM0001‐10, Guangzhou, China). MDCK cells were transfected with the siRNAs using Lipo8000 (Beyotime). The expression of indicated genes was evaluated by qPCR.

### 2.8. Next‐Generation Sequencing and Bioinformatic Analysis

Cellular RNA samples were isolated from MDCK cells with indicated different treatments. cDNA libraries were produced. Differentially expressed genes (DEGs), Gene Ontology (GO) analysis, and Kyoto Encyclopedia of Genes and Genomes (KEGG) signaling pathway analysis were performed as in the previous study [22].

### 2.9. Statistical Analysis

Relative expression levels are presented as mean ± standard deviation. The statistical significance of differences was evaluated with the unpaired Student’s *t* test or one‐way ANOVA using Prism v10.2 software (GraphPad, USA) (mean ± SD, *n* = 3,  ^∗^ *p* < 0.05,  ^∗∗^ *p* < 0.01,  ^∗∗∗^ *p* < 0.001, and  ^∗∗∗∗^ *p* < 0.0001).

## 3. Results

### 3.1. ATRA Suppressed the Replication of CIV

In our previous and other studies, it has been indicated that RIG‐I plays a role in the innate antiviral response to influenza virus infection. However, it remains unclear whether ATRA, a common vitamin A metabolite, can inhibit influenza virus replication by modulating the RLR pathway or other potential pathways. Therefore, we initially pretreated MDCK cells with ATRA for 24 h, followed by influenza virus infection. Indirect immunofluorescence results showed that ATRA significantly reduced CIV replication (Figure 1A). Additionally, by determining virus replication after treating cells with different concentrations of ATRA, both qPCR and virus titration assays demonstrated that ATRA could significantly inhibit virus replication (Figure 1B,C). Furthermore, we assessed the CC_50_ of ATRA on MDCK cells, revealing a significant decrease in cell viability when ATRA concentration exceeded 50 μM (Figure 1D). The IC_50_ of ATRA on CIV was also determined to be 97.12 nM (Figure 1E).

**Figure 1:**
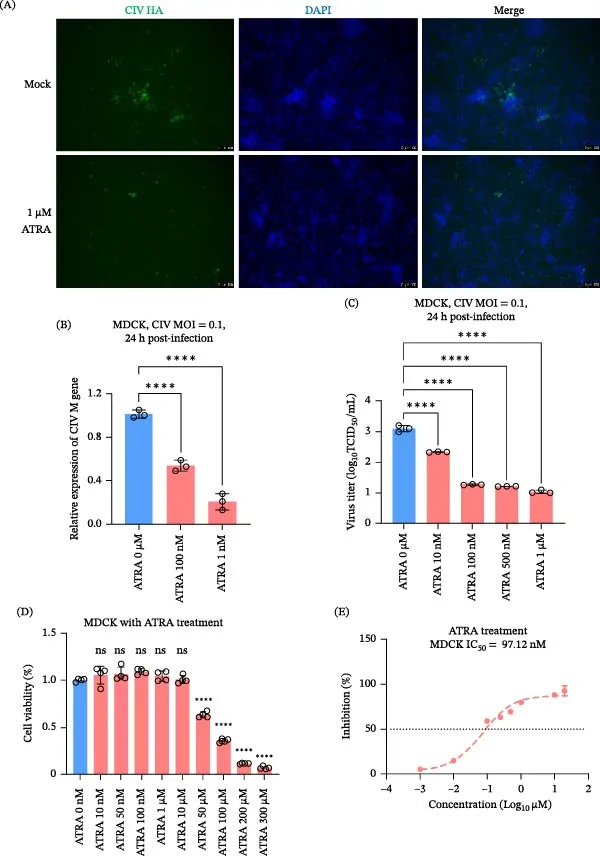
Pretreated ATRA inhibited CIV infection in MDCK cells. (A–C) IFA, qPCR, and virus titration were performed to determine the viral infection, respectively. The statistical significance of differences was evaluated with the one‐way ANOVA. Scale bar: 100 μm. (D) Cell viability assay was performed to evaluate the CC_50_. (E) IC_50_ of ATRA to inhibit CIV infection was calculated.  ^∗∗∗∗^ *p* < 0.0001. ns, no significance.

### 3.2. ATRA Does Not Inhibit CIV During the Viral Attachment and Internalization Stages

To investigate the impact of ATRA on different stages of viral replication, we initially confirmed through Western blot analysis that ATRA reduced the expression of CIV virus proteins in infected cells (Figure 2A). Based on the IC_50_ value and the above results, we selected a concentration of 1 μM ATRA for subsequent experiments. Treating cells with ATRA before, during, and after viral infection, we observed a significant decrease in CIV NP protein expression only when the treatment was applied 1 h before infection (Figure 2B). Subsequent treatment during viral attachment and internalization did not show any differences. These findings suggest that ATRA treatment might inhibit viral replication by regulating gene expression, although further experiments are needed to confirm its association with RIG‐I. Additionally, experiments on BEAS‐2B, A549, and Mv.1.Lu cells revealed that ATRA treatment did not inhibit CIV replication in human cells (BEAS‐2B and A549), while it significantly suppressed CIV replication in mink cells, similar to MDCK cells (Figure 3D,E). This indicates that the inhibitory effect of ATRA on CIV replication might be dependent on the species origin of the cells.

**Figure 2:**
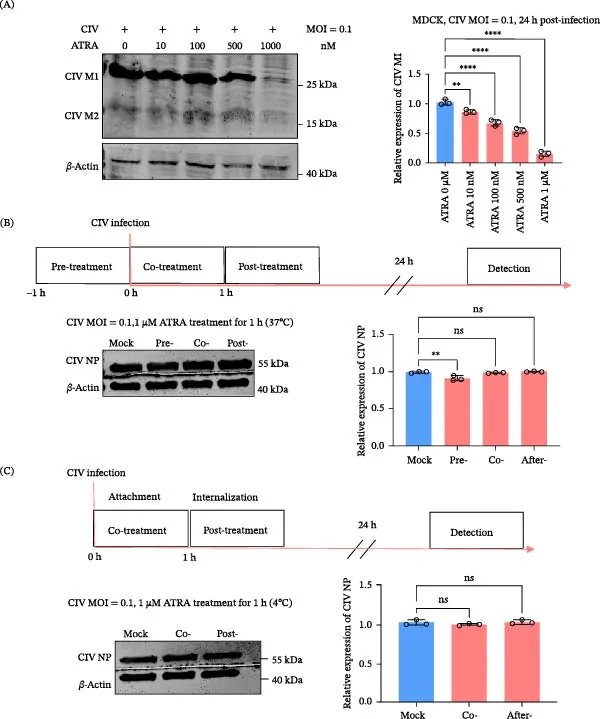
ATRA did not influence the viral attachment and internalization during the CIV infection. (A) Western blotting was performed to determine the viral infection. (B) Western blotting was performed to evaluate the antiviral effect of ATRA among pretreatment, cotreatment, and posttreatment. (C) Western blotting was performed to evaluate the antiviral effect of ATRA on viral attachment and internalization. The statistical significance of differences was evaluated with the one‐way ANOVA.  ^∗∗^ *p* < 0.01,  ^∗∗∗∗^ *p* < 0.0001. ns, no significance.

**Figure 3:**
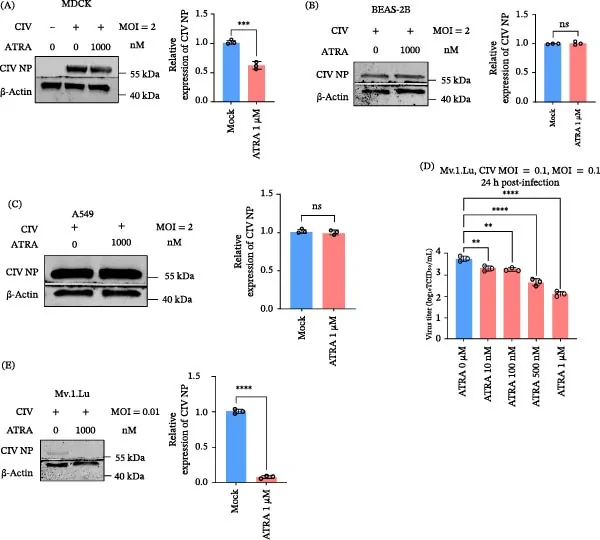
ATRA did not inhibit the CIV infection in BEAS‐2B and A549 cells. (A–C) Western blotting was performed to determine the viral infection in MDCK, BEAS‐2B, and A549 cells at high MOI, respectively. (D, E) Virus titration and western blotting were performed to determine the CIV infection in Mv.1.Lu cells. The statistical significance of differences was evaluated with the unpaired Student’s *t* test.  ^∗∗^p < 0.01,  ^∗∗∗^p < 0.001,  ^∗∗∗∗^p < 0.0001. ns, no significance.

### 3.3. Transcriptome Analysis of ATRA on MDCK Cells

To further investigate the regulatory role of ATRA in suppressing CIV replication, we utilized transcriptomic analysis. ATRA treatment alone upregulated 418 genes and downregulated 339 genes. Upon CIV infection, 2534 genes were upregulated, and 1342 genes were downregulated. A comparison of ATRA + CIV to the Mock group revealed 2686 upregulated genes and 1342 downregulated genes. In the ATRA + CIV group compared to the CIV group, 339 genes were upregulated and 315 genes were downregulated (Supporting Information 2: Figure S1). GO analysis indicated that ATRA treatment upregulated processes such as positive regulation of cell population proliferation, positive regulation of the apoptotic process, cell‐matrix adhesion, beta‐catenin binding, and extracellular region while downregulating processes including positive regulation of gene expression, negative regulation of the apoptotic process, peptidase inhibitor activity, plasma membrane, and ciliary basal body. Upon CIV infection, genes related to immune response, serine‐type endopeptidase inhibitor activity, extracellular space, and extracellular region were upregulated (Supporting Information 3: Figure S2 and Supporting Information 4: Figure S3). KEGG analysis revealed that post‐ATRA treatment, various pathways were upregulated including the PPAR signaling pathway, mitophagy, and fatty acid metabolism, while pathways like fatty acid biosynthesis, PI3K‐Akt signaling pathway, and ferroptosis were downregulated. CIV and ATRA + CIV groups each exhibited distinct pathway regulations (Supporting Information 5: Figure S4). Finally, qPCR results confirmed successful modeling in cells, showing significant upregulation of representative genes induced by ATRA and ATRA + CIV, such as CYP26B1 and RAI14, with corresponding bar graphs illustrating gene expression changes from the transcriptomic analysis (Supporting Information 6: Figure S5 and Supporting Information 7: File 6, Supporting Information 8, Supporting Information 9: File 8, Supporting Information 10: File 9).

### 3.4. ATRA‐Induced Canine KRT27 Inhibited CIV by Modulating Autophagy and RIG‐I

Further analysis revealed that following ATRA treatment of MDCK cells, the gene showing the most significant upregulation was KRT27. Notably, KRT27 gene expression was significantly upregulated in cells infected with CIV as well as in the ATRA + CIV group, with higher levels observed in the ATRA + CIV group compared to the CIV group. This observation was confirmed through qPCR analysis (Figure 4A,B). The upregulation of canine KRT27 might suggest its role in inhibiting CIV replication. Subsequently, a eukaryotic expression plasmid was constructed, and overexpression of the KRT27 gene in MDCK cells was shown to significantly suppress CIV replication, as evidenced by qPCR, viral titration, and viral protein detection (Figure 4C–E). However, the detailed mechanism by which ATRA upregulates KRT27 to suppress CIV replication remains unclear. Therefore, further experiments were conducted. Silencing of the canine KRT27 gene expression in MDCK cells using siRNA confirmed that decreased KRT27 expression promotes CIV replication following viral infection (Figure 5A). A literature review revealed that ATRA induces cellular autophagy. Interestingly, CIV is known to exploit cellular autophagy to enhance its replication, yet ATRA inhibits CIV replication in this study. This raised questions about how ATRA‐regulated autophagy impacts CIV replication and whether ATRA‐induced overexpression of canine KRT27 affects the RIG‐I signaling pathway and cellular autophagy. Subsequent experiments showed that overexpression of canine KRT27 enhanced RIG‐I protein expression (although no upregulation of the RIG‐I gene by ATRA was found in the transcriptome) and also promoted autophagy. We also observed a plasmid dose‐dependent inhibition of CIV replication by canine KRT27. Notably, high‐level expression of canine KRT27 in MDCK cells upon CIV infection led to increased expression of LC3‐II compared to noninfected conditions (Figure 5B). Additionally, experiments investigating the potential of ATRA to induce autophagy in MDCK cells revealed a significant promotion of autophagy, as evidenced by the formation of LC3‐positive autophagosomes and enhanced expression of ATG16L1 following ATRA treatment. Particularly for LC3‐positive puncta, compared with the rapamycin‐treated group, the ATRA‐treated group showed a greater promotion of LC3‐containing autophagosome formation (although no difference was observed compared with the chloroquine‐treated group) (Figure 6). These findings suggest that ATRA‐induced canine KRT27 might inhibit CIV replication by modulating innate immune response and cellular autophagy.

**Figure 4:**
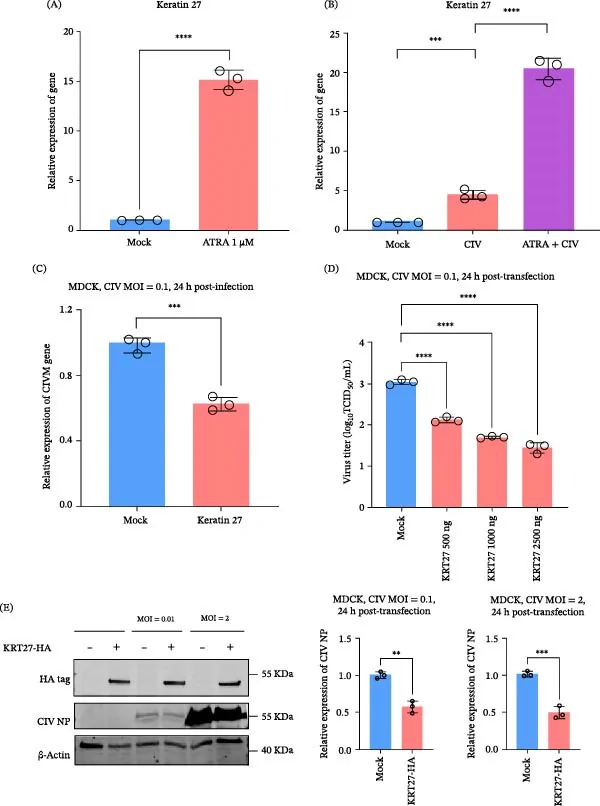
ATRA induced canine keratin 27 to suppress the CIV infection. (A) ATRA treatment increased the transcription level of canine keratin 27. (B) CIV infection triggered the transcription of canine keratin 27. (C) The overexpression of canine keratin 27 suppressed the CIV infection. (D) The antiviral effect of canine keratin 27 was dose dependent. (E) Canine keratin 27 suppressed the CIV infection at low and high MOI, respectively. The statistical significance of differences was evaluated with the unpaired Student’s *t* test or one‐way ANOVA.  ^∗∗^p < 0.01,  ^∗∗∗^p < 0.001,  ^∗∗∗∗^p < 0.0001.

**Figure 5:**
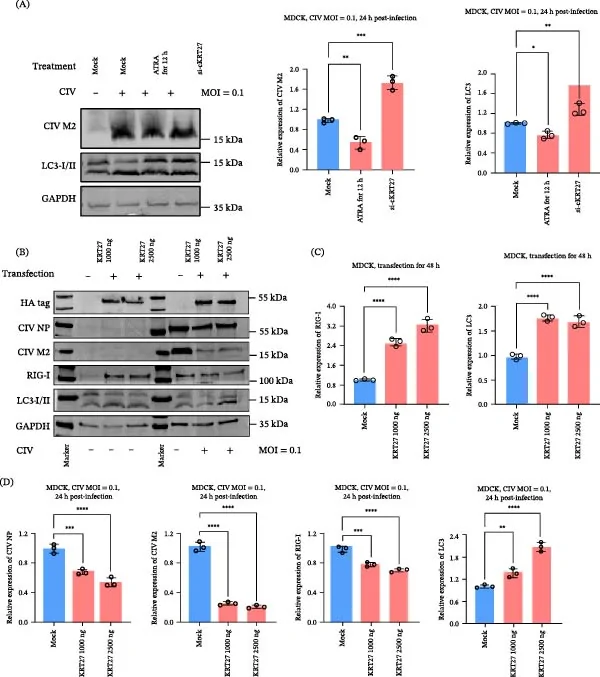
Canine keratin 27 enhanced the expression of RIG‐I and autophagy to inhibit CIV infection. (A) ATRA triggered the autophagy in CIV‐infected MDCK cells. (B–D) Quantitative analysis of canine keratin–induced regulation of RIG‐I and autophagy by western blotting assay. The statistical significance of differences was evaluated with the one‐way ANOVA.  ^∗∗^p < 0.01,  ^∗∗∗^p < 0.001, ^∗∗∗∗^p < 0.0001.

**Figure 6:**
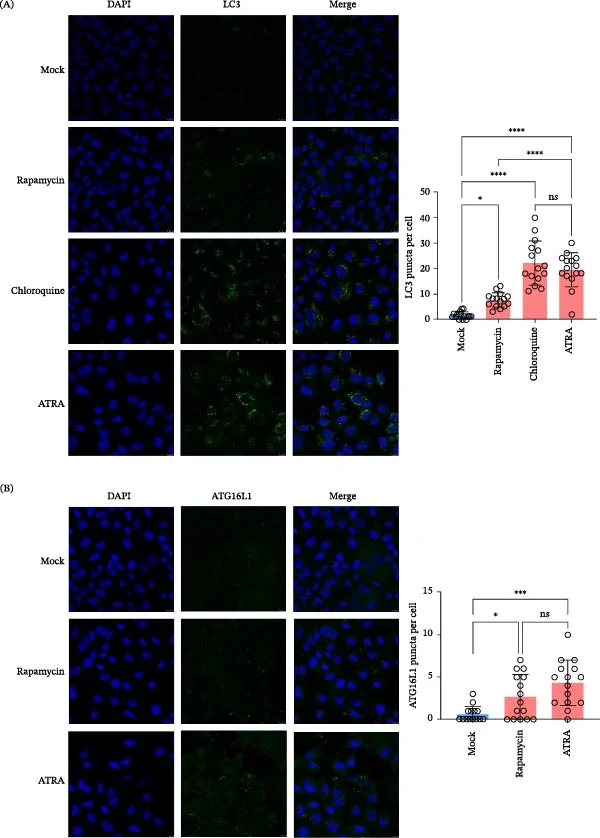
ATRA enhanced the autophagy by facilitating the production of autophagosomes. (A, B) Quantitative analysis of ATRA‐induced autophagy in MDCK cells through the IFA assay. Scale bar: 10 μm. The statistical significance of differences was evaluated with the one‐way ANOVA.  ^∗^p < 0.05,  ^∗∗∗^p < 0.001,  ^∗∗∗∗^p < 0.0001. ns, no significance.

## 4. Discussion

In previous studies, ATRA has been shown to significantly inhibit certain RNA viruses in cells. Specifically, ATRA can block the binding of ACE2 with SARS‐CoV‐2, thereby inhibiting viral replication [14]. Additionally, ATRA can activate the RIG‐I signaling pathway, enhancing the expression of type I interferons to suppress replication of mumps virus or measles virus [10, 23]. Moreover, ATRA also exhibits a certain inhibitory effect on hepatitis B virus infection [13]. However, a recent study revealed that ATRA can promote the replication of H9N2 avian influenza virus in mice, which raises questions about its effects on other subtypes of IAV [24]. In this study, we investigated the potential regulatory role of the common nutrient metabolite ATRA on the replication of CIV and confirmed its inhibitory effect on the H3N2 subtype of CIV. This suggests that ATRA might have different regulatory effects on different subtypes of IAV, indicating potential variations in the mechanisms by which ATRA regulates IAV replication. Since this study was limited to in vitro experiments, further in vivo studies are needed to confirm the consistency of these findings in both cellular and animal models. Additionally, it was noted that ATRA specifically inhibits CIV replication in cells of canine and mink origin but not in human‐derived cells. This observation might provide insight into the limited efficacy of prolonged ATRA supplementation in enhancing host defense against IAV infection in humans. Therefore, future studies are warranted to investigate the antiviral effects of ATRA against IAV infection in cells derived from different species, as well as the underlying molecular mechanisms.

In this study, we found that ATRA can significantly inhibit virus replication at low dosages. It was observed that ATRA does not inhibit CIV replication by weakening virus adsorption or internalization in infected cells but possibly by regulating gene expression within the cells. Transcriptomic analysis of ATRA‐treated MDCK cell groups revealed a significant upregulation of the KRT27 gene. Furthermore, comparing the CIV‐infected group with the ATRA + CIV group, it was found that CIV infection promotes KRT27 gene expression in MDCK cells, with the highest expression observed in the ATRA + CIV group. This suggests that KRT27 might play a crucial role during CIV infection, and ATRA might inhibit CIV replication by enhancing KRT27 gene expression. Keratins are typically known as major structural proteins in hair, contributing to cellular mechanical strength by forming intermediate filament networks that resist shear and pressure to prevent cell rupture [25, 26]. Additionally, keratins have been implicated in stress responses, cell survival, and interactions with mitochondria [27, 28]. Recent studies have shown that keratin 8 is involved in virus replication processes, playing diverse roles in IAV replication [29, 30]. Moreover, keratin 10 has been found to inhibit the release of the genomic material of H9N2 avian influenza virus in infected cell endosomes [31]. These studies collectively demonstrate the impact of keratin proteins on virus replication, with our discovery of KRT27 adding further insights into this understanding.

In this study, we observed that the overexpression of KRT27 alone (in the absence of infection) can promote the expression of cellular RIG‐I and regulate autophagy in cells. Interestingly, transcriptomic data showed that ATRA did not promote the RIG‐I gene expression in MDCK cells, whereas Western blot analysis revealed that ATRA upregulated the RIG‐I protein expression. This suggests that ATRA may control the RIG‐I protein expression by regulating its posttranslational modifications in canine‐derived cells. Under infection conditions and at the highest transfection dose, cellular autophagy is significantly enhanced compared to other conditions, suggesting that the main mechanism by which KRT27 inhibits viral replication might be through the suppression of viral replication via autophagy. Additionally, we investigated the status of cellular autophagy under ATRA‐treated cell conditions and found that ATRA indeed significantly enhances cellular autophagy, thereby indirectly confirming our speculation. During the CIV infection with KRT27 transfection, the expression of the RIG‐I protein decreases, possibly due to KRT27 inhibiting viral replication, leading to a weakened response of RIG‐I. This phenomenon may be attributed to the interaction between RIG‐I and viral components during the period when KRT27 simultaneously enhances RIG‐I expression and autophagosome formation, leading to the incorporation of RIG‐I into autophagosomes for degradation. Previous studies have indicated that autophagy plays a dual role in the replication of the influenza virus. The virus can hijack autophagy to promote its own replication by mechanisms such as avian influenza virus M2 inhibiting the interaction between RAB7 and TBC1D5, thereby suppressing the host cell’s degradation of viral proteins [32]. Additionally, the virus utilizes RAB33B‐TBC1D25 to facilitate M2 intracellular trafficking for enhanced replication [22], and PB1, PB1‐F2, and M2 proteins can modulate innate immunity through autophagy to promote viral replication [33–35]. Furthermore, host autophagy can degrade viral components through both classical and nonclassical autophagy pathways [36, 37]. Increased LC3 puncta/LC3‐II and ATG16L1 were observed, consistent with altered autophagosome dynamics; flux‐inhibitor experiments would be needed to distinguish increased formation from impaired clearance. These studies underscore the complexity of the interaction between host cell autophagy and the influenza virus, with different outcomes depending on which aspect predominates.

In conclusion, this study proposed that ATRA and ATRA‐induced KRT27 might modulate autophagy and innate immune response by a novel molecular mechanism, consequently inhibiting virus‐induced autophagic manipulation (Figure 7). This proposed mechanism is anticipated to represent a novel research trajectory, offering a pivotal groundwork for the advancement of new therapeutic strategies targeting influenza virus infection.

**Figure 7:**
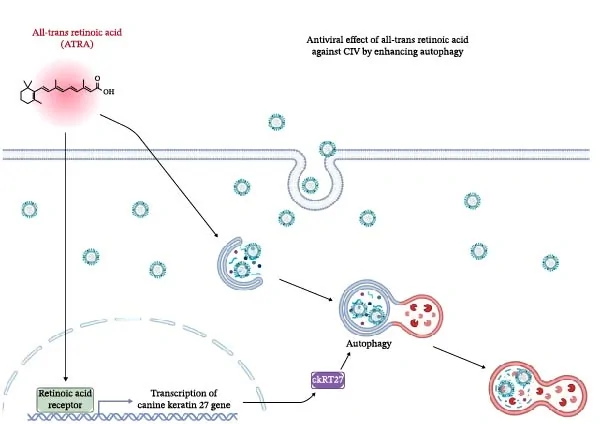
Schematic diagram of potential mechanism of ATRA against CIV infection through keratin 27–mediated modulation.

## Author Contributions

**Qi Li**: conceptualization, investigation, visualization, writing – original draft. **Shaotang Ye**: data curation, formal analysis, validation, writing – review and editing. **Qi Jiang**: software, writing – review and editing. **Benqiang Li**: resources. **Jinghua Cheng**: investigation, writing – original draft. **Jie Tao**: methodology, resources. **Pan Tang**: methodology. **Ying Shi**: writing – review and editing. **Huili Liu**: funding acquisition, resources, writing – review and editing.

## Funding

This study was supported by the SAAS Program for Excellent Research Team (Grants [2022]012 and [2025]026).

## Ethics Statement

The authors have nothing to report.

## Consent

The authors have nothing to report.

## Conflicts of Interest

The authors declare no conflicts of interest.

## Supporting Information

Additional supporting information can be found online in the Supporting Information section.

## Supporting information


**Supporting Information 1** File 10: Antibodies and primer sets used in this study were listed.

**Supporting Information 2** Figure S1: Differentially expressed gene (DEG) analysis of the four different groups.

**Supporting Information 3** Figure S2: GO analysis of the ATRA vs. Mock and CIV vs. Mock groups.

**Supporting Information 4** Figure S3: GO analysis of the ATRA + CIV vs. Mock and ATRA + CIV vs. CIV groups.

**Supporting Information 5** Figure S4: KEGG pathway analysis of the four different groups.

**Supporting Information 6** Figure S5: Partial ATRA‐induced genes without CIV infection were shown.

**Supporting Information 7** File 6: DEGs of ATRA vs. Mock.

**Supporting Information 8** DEGS of CIV vs. Mock.

**Supporting Information 9** File 8: DEGs of ATRA + CIV vs. Mock.

**Supporting Information 10** File 9: DEGs of ATRA + CIV vs. CIV.

## Data Availability

The data that support the findings of this study are available from the corresponding author upon reasonable request.
