# Supplementary material for: All‐Trans Retinoic Acid Inhibits Canine Influenza Virus Replication Through Keratin 27–Mediated Autophagy Modulation
Source: Transbound Emerg Dis. 2026 Oct 22;2026:2088414. doi: 10.1155/tbed/2088414 (PMC13687935; doi:10.1155/tbed/2088414)
Supplement: Supplementary file 1 [file TBED-2026-2088414-s009.docx]

**Table S1** Primer pairs used for quantitative RT-PCR analysis.

| **Primer** | **Sequence of oligonucleotides (5′ → 3)** |
| --- | --- |
| Canine KRT27-qF | AGCTCCTCGACATCAAGGTCCA |
| Canine KRT27-qR | GCCACGAGGATCTATTTCTTCAACA |
| Canine GAPDH-qF | AATTCCACGGCACAGTCAAGGC |
| Canine GAPDH-qR | ACAACATACTCAGCACCAGCATCAC |
| CIV-M-qF | TGATCCTCTCGTTATTGCCGCAAG |
| CIV-M-qR | CACTCTGCTGTTCCTGCCGATAC |

**Table S2** Antibody used in this study

| **Antibody** | **Company** | **Host species** | **Catalog number** |
| --- | --- | --- | --- |
| RIG-I | Millipore | Mouse | MABF297 |
| LC3B | Abcam | Rabbit | AB192890 |
| ATG16L1 | Abcam | Rabbit | AB187671 |
| HA tag | R&D | Rabbit | NB600-363 |
| IAV HA H3N2 | GenteTex | Rabbit | GTX127363 |
| IAV M1 | GeneTex | Rabbit | GTX125928 |
| IAV M2 | GenteTex | Rabbit | GTX125951 |
| IAV NP | GenteTex | Rabbit | GTX125989 |
| β-actin | GenteTex | Rabbit | GTX109639 |
| GAPDH | GenteTex | Rabbit | GTX100118 |
| Alexa Fluor® 594 | Abcam | Goat | AB150077 |
| Alexa Fluor® 680 | Abcam | Goat | AB175772 |
| Alexa Fluor® 790 | Abcam | Goat | AB175783 |
